# Supplementary material for: A galanin-positive population of lumbar spinal cord neurons modulates sexual arousal and copulatory behavior in male mice
Source: Nat Commun. 2025 Sep 23;16:8282. doi: 10.1038/s41467-025-63877-2 (PMC12457683; doi:10.1038/s41467-025-63877-2)
Supplement: Supplementary file 2 — Description of Additional Supplementary Files [file 41467_2025_63877_MOESM2_ESM.pdf]

Description of Additional Supplementary Files

Supplemental Movie 1
Light evoked BSM-MN activity leads to characteristic pelvic floor movements

Supplemental Movie 2
Pelvic floor movements during ejaculation in a sexually behaving male mouse

Supplemental Movie 3
Conservation of erection behavior in a DTR animal.

Supplemental Movie 4
Erection behavior in a SHAM animal.
