# Supplementary material for: A galanin-positive population of lumbar spinal cord neurons modulates sexual arousal and copulatory behavior in male mice
Source: Nat Commun. 2025 Sep 23;16:8282. doi: 10.1038/s41467-025-63877-2 (PMC12457683; doi:10.1038/s41467-025-63877-2)
Supplement: Supplementary file 7 — Reporting Summary [file 41467_2025_63877_MOESM7_ESM.pdf]

nature portfolio

Corresponding author(s): Constanze Lenschow, Susana Q. Lima

Last updated by author(s): Jun 20, 2025

Reporting Summary

Nature Portfolio wishes to improve the reproducibility of the work that we publish. This form provides structure for consistency and transparency in reporting. For further information on Nature Portfolio policies, see our Editorial Policies and the Editorial Policy Checklist.

Statistics

For all statistical analyses, confirm that the following items are present in the figure legend, table legend, main text, or Methods section.

n/a	Confirmed	
	x	The exact sample size (*n*) for each experimental group/condition, given as a discrete number and unit of measurement
	x	A statement on whether measurements were taken from distinct samples or whether the same sample was measured repeatedly
	x	The statistical test(s) used AND whether they are one- or two-sided<br>*Only common tests should be described solely by name; describe more complex techniques in the Methods section.*
x		A description of all covariates tested
	x	A description of any assumptions or corrections, such as tests of normality and adjustment for multiple comparisons
	x	A full description of the statistical parameters including central tendency (e.g. means) or other basic estimates (e.g. regression coefficient) AND variation (e.g. standard deviation) or associated estimates of uncertainty (e.g. confidence intervals)
	x	For null hypothesis testing, the test statistic (e.g. F, t, r) with confidence intervals, effect sizes, degrees of freedom and P value noted<br>*Give P values as exact values whenever suitable.*
x		For Bayesian analysis, information on the choice of priors and Markov chain Monte Carlo settings
x		For hierarchical and complex designs, identification of the appropriate level for tests and full reporting of outcomes
x		Estimates of effect sizes (e.g. Cohen's *d*, Pearson's *r*), indicating how they were calculated

Our web collection on statistics for biologists contains articles on many of the points above.

Software and code

Policy information about availability of computer code

Data collection	Eletrophysiology data were amplified with a Dagan BVC-700A amplifier (Dagan, Minneapolis, MN), sampled by a data-acquisition interface (Power 1401, CED, Cambridge, England) and controlled and analyzed by the spike2 software (CED, Cambridge, England).<br>In vivo BSM recordings behavioral experiments were recorded using two point gray cameras (Teledyne FLIR) controlled using Bonsai Visual Reactive Programming.<br>In vivo BSM EMG recordings: RHD 16-Channel bipolar-input recording headstage (Intan Technologies), connected to an Acquisition Board (Open Ephys), using a sampling rate of 30KHz. The board and cameras were controlled using a Bonsai script (Bonsai Visual Reactive Programming).<br>Spinal cord sections were imaged using a Slide Scanner (Zeiss AxioScan.Z1, Zeiss Microscopy).
Data analysis	Matlab 2021a and 2024a. Spyder 3.3.6 (Python). Julia v1.11.1. Zen Software (Zen 2.6, Zeiss Microscopy). Spike2 software (CED, Cambridge, England). Videos were analyzed using Python Video Annotator (developed at the Champalimaud Foundation).

For manuscripts utilizing custom algorithms or software that are central to the research but not yet described in published literature, software must be made available to editors and reviewers. We strongly encourage code deposition in a community repository (e.g. GitHub). See the Nature Portfolio guidelines for submitting code & software for further information.

Data

Policy information about availability of data

All manuscripts must include a data availability statement. This statement should provide the following information, where applicable:

- Accession codes, unique identifiers, or web links for publicly available datasets
- A description of any restrictions on data availability
- For clinical datasets or third party data, please ensure that the statement adheres to our policy

All data reported in the current manuscript can be found in the following Mendeley data repository DOI: . https://data.mendeley.com/datasets/xyh6wwd7fz/1. Source data files are provided for this paper. Any further request can be addressed to the corresponding authors (C.L and S.Q.L.).

Research involving human participants, their data, or biological material

Policy information about studies with human participants or human data. See also policy information about sex, gender (identity/presentation), and sexual orientation and race, ethnicity and racism.

Reporting on sex and gender	n/a
Reporting on race, ethnicity, or other socially relevant groupings	n/a
Population characteristics	n/a
Recruitment	n/a
Ethics oversight	n/a

Note that full information on the approval of the study protocol must also be provided in the manuscript.

Field-specific reporting

Please select the one below that is the best fit for your research. If you are not sure, read the appropriate sections before making your selection.

☒ Life sciences ☐ Behavioural & social sciences ☐ Ecological, evolutionary & environmental sciences

For a reference copy of the document with all sections, see nature.com/documents/nr-reporting-summary-flat.pdf

Life sciences study design

All studies must disclose on these points even when the disclosure is negative.

Sample size	No statistical methods were used to predetermine sample sizes. Sample sizes were based on previously published studies in the field and are consistent with those generally used in similar experimental designs. See: Truitt and Coolen, Science 2002 and Qi L. et al., Nature 2024.
Data exclusions	There was no data excluded from the experiments conducted in this study.
Replication	All experiments presented in this manuscript include multiple independent biological replicates (N mice), as detailed in the figure legends and Methods section. For the majority of core findings, independent replicates were conducted by multiple experimenters to ensure robustness and reproducibility. In addition, most datasets were independently analyzed by multiple experimenters, and all analyses were performed blind to experimental conditions. While each full experiment (i.e., a complete set of mice for a given condition) was not repeated in its entirety more than once, the main findings have been confirmed across these independently conducted replicates.
Randomization	All experimental subjects were randomly allocated into experimental groups.
Blinding	All manual analysis was performed blind to the experimental conditions as stated in the methods of this manuscript.

Reporting for specific materials, systems and methods

We require information from authors about some types of materials, experimental systems and methods used in many studies. Here, indicate whether each material, system or method listed is relevant to your study. If you are not sure if a list item applies to your research, read the appropriate section before selecting a response.

Materials & experimental systems

n/a	Involved in the study
	☒ Antibodies
☒	Eukaryotic cell lines
☒	Palaeontology and archaeology
	☒ Animals and other organisms
☒	Clinical data
☒	Dual use research of concern
☒	Plants

Methods

n/a	Involved in the study
☒	ChIP-seq
☒	Flow cytometry
☒	MRI-based neuroimaging

Antibodies

Antibodies used	Gastrin Releasing Peptide (GRP) (Porcine) - Antibody, Phoenix Pharmaceuticals, H-027-13, Lot# 01742-1).<br>Substance P (Anti-Substance P Receptor Antibody, Sigma-Aldrich, AB15810, Lot# 3022869).<br>Enkephalin (Anti-Enkephalin/ENK antibody, Abcam, ab85798).<br>CCK (Polyclonal Rabbit anti-Human CCK / Cholecystokinin Antibody, LSBio, LS-C190673, aa26-33).<br>Osteopontin (Mouse Osteopontin/OPN Antibody, R&D Systems, AF808, Lot# BDO0617401).<br>VGLUT1 (VGLUT 1 antibody, Synaptic Systems, 135 304, Q62362).<br>Galanin (Anti-Galanin Antibody, Milipore, AB2233, Lot# 3096488).<br>Rabbit anti-cFos, Synaptic Systems, 226 003, Rb108B5).
Validation	Gastrin Releasing Peptide (GRP) (Porcine) - Antibody: 100% Cross-reactivity with control Gastrin Releasing Peptide (Porcine) and Gastrin Releasing Peptide (Human) (reported by provider: http://www.phoenixbiotech.net/catalog/product_info.php?products_id=6602).<br>Anti-Substance P Receptor Antibody: Not externally validated.<br>Anti-Enkephalin/ENK antibody: Pre-absorption with Human Enkephalin/ENK peptide - ab102738 (reported by provider).<br>Polyclonal Rabbit anti-Human CCK: Not externally validated.<br>Anti-Vglut1: K.O. verified (reported by provider).<br>Mouse anti Osteopontin/OPN Antibody: Pre-absorption with Recombinant Mouse Osteopontin/OPN Protein (Catalog # 441-OP) (reported by provider).<br>Anti-Galanin Antibody: Expression on rat cerebellum tissue consistent with galanin specific detection (reported by provider).<br>Rabbit anti-cFos: Pre-absorption with c-Fos peptide - 226-0P (reported by provider).

Animals and other research organisms

Policy information about studies involving animals; ARRIVE guidelines recommended for reporting animal research, and Sex and Gender in Research

Laboratory animals	This study focused on the mouse (Mus musculus)<br>A small cohort of Long Evans rats were also used (N=3)
Wild animals	This study did not involve wild animals.
Reporting on sex	Findings of the current study are focused on the circuits for ejaculation and therefore only apply to male subjects.
Field-collected samples	This study did not involve field collected samples
Ethics oversight	All experimental procedures were approved by the Animal Care and Users Committee of the Champalimaud Neuroscience Program, the Portuguese National Authority for Animal Health (Direcção Geral de Veterinária; approval number 0421/000/000/2022) and by the local ethic committee of the University of Bordeaux and the French Agriculture and Forestry Ministry for handling animals (approval number 2016012716035720).

Note that full information on the approval of the study protocol must also be provided in the manuscript.

Plants

Seed stocks	n/a
Novel plant genotypes	n/a
Authentication	n/a
